# Ranking Enzyme Structures in the PDB by Bound Ligand Similarity to Biological Substrates

**DOI:** 10.1016/j.str.2018.02.009

**Published:** 2018-04-03

**Authors:** Jonathan D. Tyzack, Laurent Fernando, Antonio J.M. Ribeiro, Neera Borkakoti, Janet M. Thornton

**Affiliations:** 1 EMBL-EBI, Wellcome Genome Campus, Hinxton CB10 1SD, UK

**Keywords:** enzyme, PDB, similarity, ligand, biological, relevance, bound, cognate, native

## Abstract

There are numerous applications that use the structures of protein-ligand complexes from the PDB, such as 3D pharmacophore identification, virtual screening, and fragment-based drug design. The structures underlying these applications are potentially much more informative if they contain biologically relevant bound ligands, with high similarity to the cognate ligands. We present a study of ligand-enzyme complexes that compares the similarity of bound and cognate ligands, enabling the best matches to be identified. We calculate the molecular similarity scores using a method called PARITY (proportion of atoms residing in identical topology), which can conveniently be combined to give a similarity score for all cognate reactants or products in the reaction. Thus, we generate a rank-ordered list of related PDB structures, according to the biological similarity of the ligands bound in the structures.

## Highlights

• We present PARITY, matching atoms in identical topology to gauge ligand similarity
• Bound-cognate ligand similarity is a useful metric for ranking PDB structures
• Only 26% of enzyme structures in the PDB have bound-cognate ligand similarity ≥0.7
• We provide rank-ordered lists of PDBs with the most biologically relevant ligands

## Graphical Abstract

**Figure:**
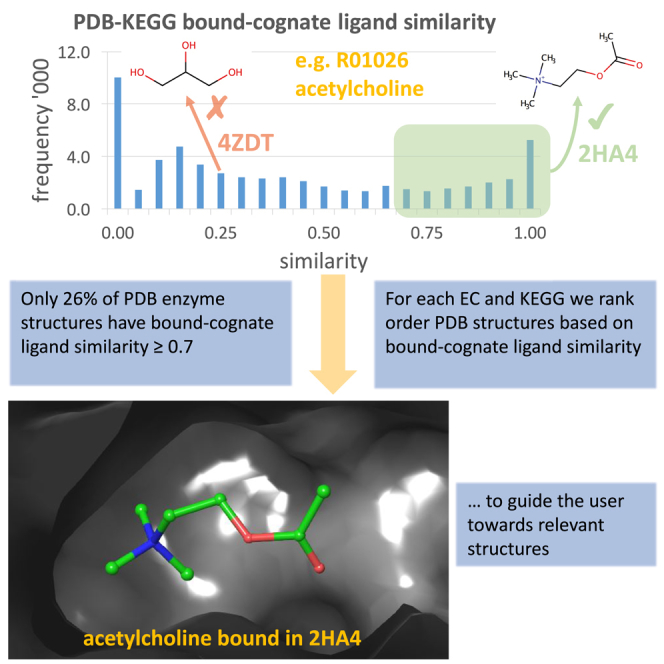

## Teaser

Tyzack et al. present a similarity study, comparing bound ligands in enzyme structures deposited in the PDB with biological substrates in native reactions. They identify structures most likely to contain relevant information regarding enzyme-substrate interactions and binding interfaces.

## Introduction

Enzymes are an important group of drug targets where understanding ligand-enzyme binding requires inspection of crystal structures with bound ligands. The ligand-enzyme complex becomes more informative if the bound ligand is similar to the cognate ligand (i.e., the compound expected to bind *in vivo*), allowing the binding site and ligand-enzyme interactions to be identified more completely. With unmodified target proteins, it is often not possible to bind cognate ligands without the reaction occurring, so compounds with varying degrees of similarity to the cognate ligands are used as surrogates in co-crystallization experiments.

There are many databases of ligand-protein binding sites, which aim to define the binding cavity and identify important protein-ligand interactions (Stuart et al., 2002, Hendlich et al., 2003, Golovin, 2004, Ivanisenko, 2004, Shoemaker et al., 2010, Kufareva et al., 2012, Desaphy et al., 2015), overlay the binding site with known bound ligands (Laskowski, 2004, Lombard et al., 2014), and score structures using binding affinity and resolution data (Hu et al., 2005). One challenge is to identify the best PDB (Gutmanas et al., 2014) entry for analysis, since many proteins have multiple structures available, with many different ligands. “Best” structures are often selected using their resolution, without regard for the nature of the ligands bound. For enzymes, we propose the similarity of bound and cognate small-molecule ligands as another important measure for scoring structures, where pocket identification and description would be enhanced from understanding the biological relevance of the bound substrates.

Bound protein-ligand structures are used in many applications, including virtual screening and 3D pharmacophore identification. For example, ligand-homology modeling (Drwal and Griffith, 2013) uses binding site alignment and ligand transposition (Konc et al., 2015) as the basis to score and validate protein-ligand interactions (Najmanovich et al., 2008, Shin et al., 2011, Evangelidis et al., 2012, Konc et al., 2012, Kurbatova et al., 2013, Zhou and Skolnick, 2013, Heo et al., 2014, Konc and Janežič, 2014, Cleves and Jain, 2015, Roy et al., 2015). Docking methods have also been enhanced by using the location of bound ligands to supplement scoring functions (Stanton et al., 2015, Anighoro and Bajorath, 2016) and to enable false positives to be pruned from virtual screening (Bietz et al., 2016). Large-scale computational methods that identify 3D binding pharmacophores (Meslamani et al., 2012) or represent ligand-protein interactions as networks (Kalinina et al., 2011, Martínez-Jiménez and Marti-Renom, 2015, Kasahara and Kinoshita, 2016) are also likely to be enhanced with knowledge about the biological relevance of the ligands on which they are based, potentially improving the prediction of ligand-protein interactions (Kinnings and Jackson, 2011) and the performance of machine learning methods to classify actives from decoys (Chupakhin et al., 2013).

A further example of the use of bound structures is fragment-based design, which links fragments from bound substrates to design ligands (Desaphy and Rognan, 2014, Tang and Altman, 2014, Wang et al., 2015). In many cases the bound ligands are inhibitors or are molecules with their active fragments modified, so a guide to find those ligands that are most similar to the cognate molecule could help in the design of active molecules.

**Figure 1:**
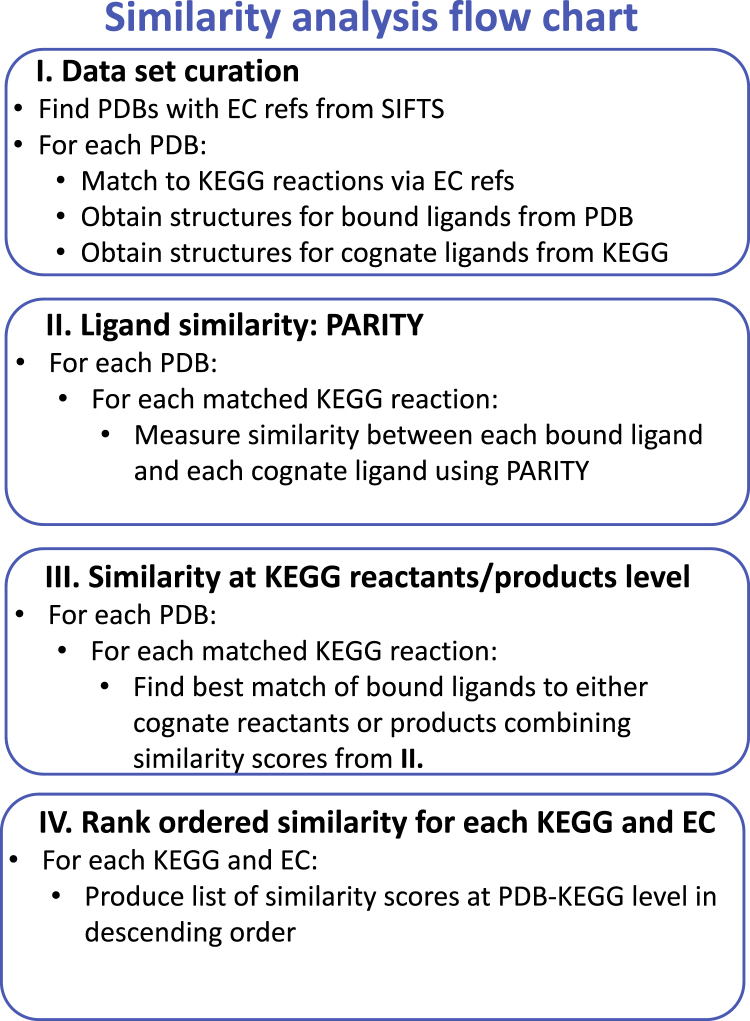
Flowchart to Summarize the Overall Methodology

**Figure 2:**
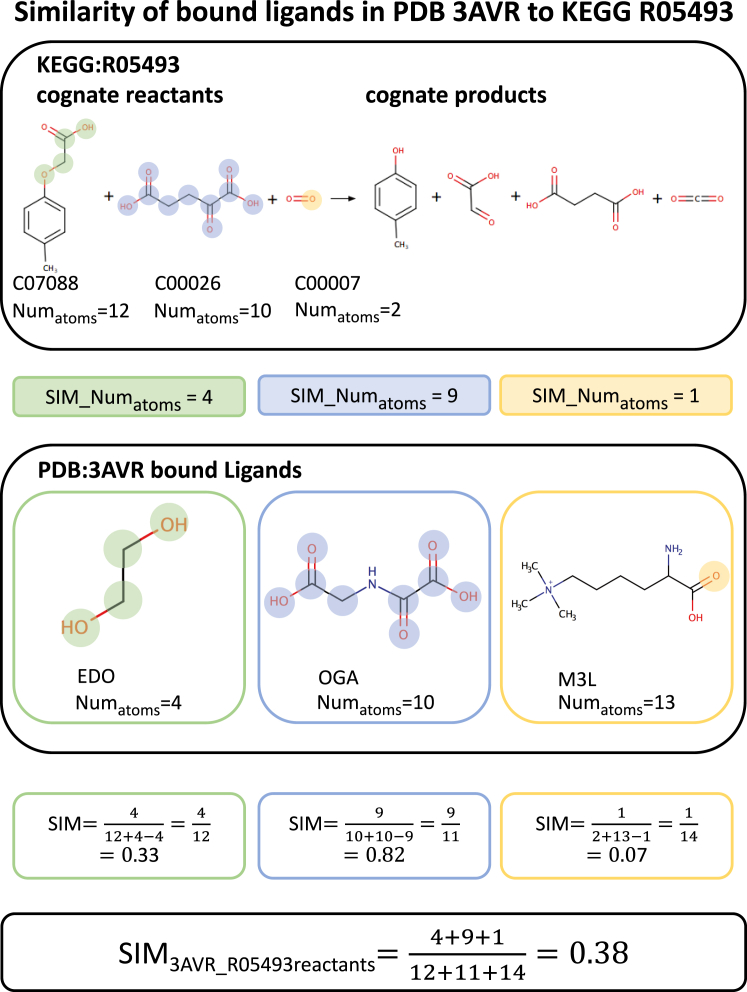
PARITY Example Illustration of the similarity calculation for KEGG R05493 (alpha-ketoglutarate-dependent 2,4-dichlorophenoxyacetate dioxygenase) and PDB: 3AVR (catalytic fragment of UTX/KDM6A bound with histone H3K27me3 peptide, N-oxyalylglycine, and Ni(II)). The cognate reactants C07088 (4-chlorophenoxyacetate), C00026 (2-oxoglutarate), and C00007 (oxygen) are matched to the most similar bound ligands (OGA [N-oxalylglycine], EDO [1,2-ethanediol], and M3L [N-trimethyllysine], respectively) using PARITY by matching atoms of the same type in equivalent topological positions. The 2D ligand graphics were generated using MarvinSketch (ChemAxon, 2016).

Herein, we present a method called PARITY (proportion of atoms residing in identical topology) to compare the similarity of bound and cognate ligands and automatically annotate the current content of the PDB. The methodology is described in detail in the online STAR Methods and summarized in the flow chart in Figure 1, with an example of the PARITY method provided in Figure 2. We anticipate that our similarity scores will allow researchers to identify the most representative and biologically relevant PDB structures when collating datasets for the diverse methods applying these data. The similarity scores generated from this analysis are released in the public Mendeley Data Repository.

## Results

**Figure 3:**
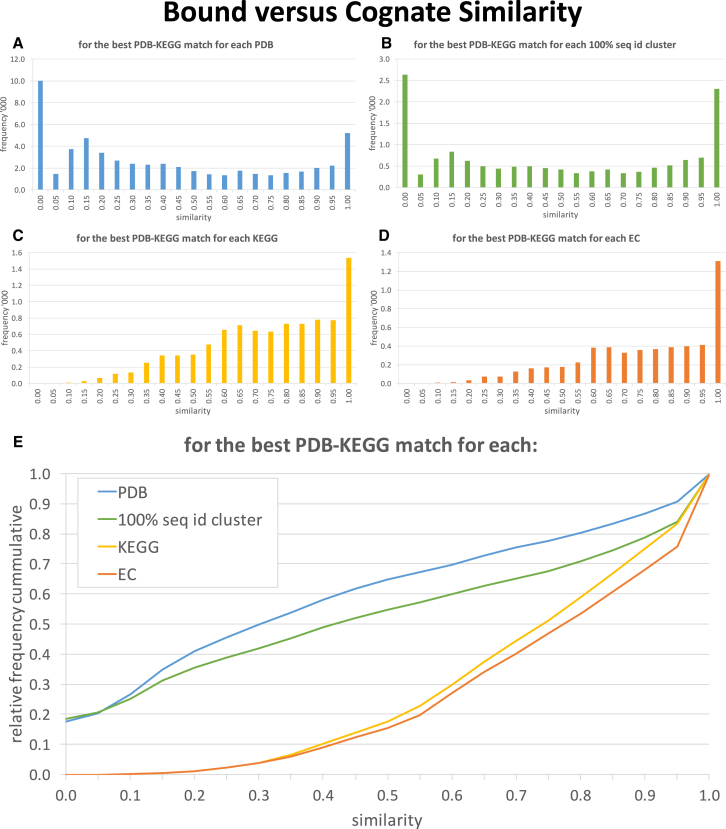
Bound Ligands versus Cognate Ligands Similarity Frequency Graphs The graphs show the frequency of the binned similarity scores of bound ligands from PDB structures and cognate ligands from KEGG reactions. Graphs are produced for the most similar PDB-KEGG match for each (A) PDB structure, (B) 100% sequence identity cluster, (C) KEGG reaction, and (D) EC reference, with relative cumulative frequency comparisons provided in (E).

In this section, we present the analyses of the similarity of bound ligands to cognate ligands across the dataset (Figure 3). Separate plots are generated finding the most similar PDB-KEGG match for each (1) PDB structure, (2) 100% sequence identity cluster, (3) KEGG reaction, and (4) EC reference represented in the dataset, with a relative frequency comparison in Figure 3E. It would be possible to cluster the sequences using looser clustering criteria, which would produce fewer clusters and would in all likelihood remove some of the poorer matches, but only the 100% sequence identity results are presented here. Summary level data are presented in Table 1 showing the percentage of the best PDB-KEGG matches in different similarity categories.

**Table 1:** Similarity Results

Best PDB-KEGG Match for Each:	Similarity Category				
	Num	None Bound, %	Sim ≤ 0.3, %	0.3 < sim < 0.7, %	Sim ≥ 0.7, %
(a) PDB	56,994	16.9	31.3	25.8	26.0
(b) Cluster	14,257	17.8	23.2	23.2	36.2
(c) KEGG	9,308	0.0	3.1	38.0	58.9
(d) EC	5,392	0.0	3.0	34.1	62.9

Table shows the number of matches and the percentage with no bound ligands, similarity ≤ 0.3, similarity between 0.3 and 0.7, and similarity ≥ 0.7 for the most similar PDB-KEGG match for each (a) PDB, (b) 100% sequence identity cluster, (c) KEGG reaction, and (d) EC.

A key observation is the high level of PDB structures where there are no bound ligands or that have bound ligands with low similarity to cognate ligands. It can be seen from Table 1 that 16.9% (9,612) of the PDBs in the study do not have bound ligands and, after finding the most similar KEGG reaction for those that do, 31.3% (17,833) have a similarity score of less than or equal to 0.3. Only 26.0% (14,839) have a similarity score of greater than or equal to 0.7.

Selecting the most similar PDB-KEGG match for each cluster of 100% sequence identity improves the situation since many of the poorer matches can be discarded. This improves further by selecting the most similar PDB-KEGG match for each KEGG and EC, where the proportion with a similarity score greater than or equal to 0.7 increases to 58.9% and 62.9%, respectively. This emphasizes the usefulness of the similarity measure and how it can be used to identify PDB structures with bound ligands most similar to the cognate ligands.

To further demonstrate the value of this dataset, we provide two use cases where it is required to find PDB structures with the most similar bound ligands, first for a particular KEGG reaction and second for a particular EC reaction. In these examples, we are able to identify the best matches from a large number of structures with varying degrees of similarity, eliminating the need to manually sift through the data and enabling research effort to be focused on inspecting the ligand binding or other higher value activities.

### Use Case 1: Finding PDBs with the Most Similar Bound Ligands to KEGG R01026

**Figure 4:**
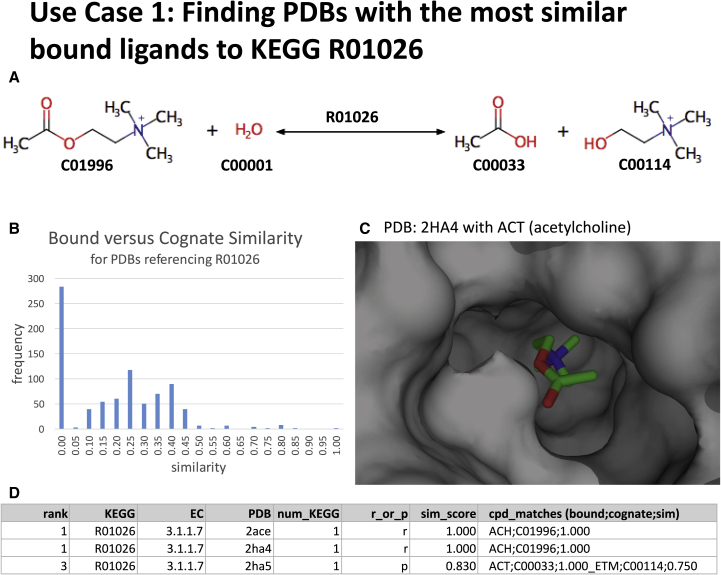
Use Case 1 Graphic shows (A) the KEGG reaction for R01026 (acetylcholine acetylhydrolase); (B) the distribution of similarity scores between the cognate ligands and the bound ligands in PDBs referencing R01026; (C) a representation of the binding pocket of the one of the best matches, PDB: 2HA4 (crystal structure of mutant S203A of mouse acetylcholinesterase complexed with acetylcholine), with bound acetylcholine; and (D) an extract from the Mendeley Data Repository for R01026. (In D num_KEGG refers to the number of KEGG reactions potentially matched to the PDB via the EC in SIFTS, r_or_p refers to whether reactants [r] or products [p] have been matched, and cpd_matches details the bound and cognate matched ligands in the format bound; cognate; similarity_score with multiple matches separated by an underscore.)

The number of PDBs referencing R01026 (acetylcholine acetylhydrolase) via its parent EC references 3.1.1.7 (acetylcholinesterase) and 3.1.1.8 (cholinesterase) is 840, with the frequency distribution of binned similarity scores shown in Figure 4B. By measuring the similarity of bound ligands in each PDB to the reactants and products in R01026 we are able ascertain that there are only 16 structures (1.9%) with a similarity score greater than or equal to 0.7 and only two exact matches. One of the exact matches, PDB: 2HA4 (crystal structure of mutant S203A of mouse acetylcholinesterase complexed with acetylcholine), is shown in Figure 4C.

### Use Case 2: Finding PDBs with the Most Similar Bound Ligands to EC 4.2.1.75

**Figure 5:**
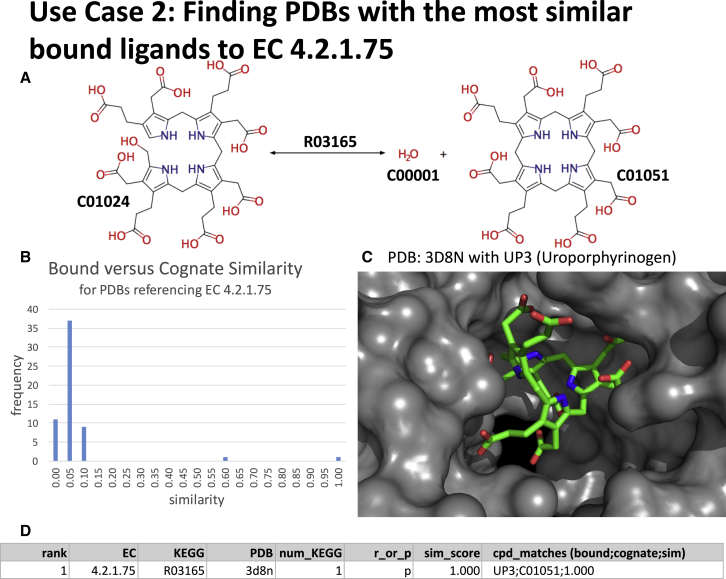
Use Case 2 Graphic shows (A) the KEGG reaction for R03165 (hydroxymethylbilane hydro-lyase [cyclizing]); (B) the distribution of similarity scores between the cognate ligands and the bound ligands in PDBs referencing EC 4.2.1.75 (uroporphyrinogen III synthase); (C) a representation of the binding pocket of the best match, PDB: 3D8N (uroporphyrinogen III synthase-uroporphyrinogen III complex), with bound uroporphyrinogen; and (D) an extract from the Mendeley Data Repository for EC 4.2.1.75. (In D num_KEGG refers to the number of KEGG reactions potentially matched to the PDB via the EC in SIFTS, r_or_p refers to whether reactants [r] or products [p] have been matched, and cpd_matches details the bound and cognate matched ligands in the format bound; cognate; similarity_score with multiple matches separated by an underscore.)

The number of PDBs referencing EC 4.2.1.75 (uroporphyrinogen III synthase) is 59, with the frequency distribution of binned similarity scores shown in Figure 5B. By measuring the similarity of bound ligands in each PDB to the reactants and products in R03165 (hydroxymethylbilane hydro-lyase [cyclizing]) we are able to ascertain that there is only one structure (1.7%) with a similarity score greater than or equal to 0.7, which is also an exact match, shown in Figure 5C.

### Validation Using Manually Curated M-CSA Dataset

A limitation of the methodology described is the potentially imprecise matching of PDB to KEGG via the EC reference number(s) in SIFTS. In many cases a PDB is uniquely matched to one KEGG reaction via its sole EC reference (38.8% of our dataset are uniquely mapped to one KEGG reaction in this way), but in some cases multiple EC numbers are listed, the EC number is partial, or the EC number maps to multiple KEGG reactions. In these cases, it is not possible to know precisely which KEGG reaction(s) is the “correct” match for that structure; some KEGG reactions may be catalyzed by the enzyme, but with lower efficiency, and some may not occur at all. This is a problem we do not attempt to resolve here; rather we explicitly show in the output data the number of KEGG reactions associated with each PDB structure so it is obvious to the user how many KEGG reactions have been matched, from which one will be selected.

It is interesting to note that 83.2% of the full PDB dataset map to only one EC number and so any related KEGG reactions can be expected to share similar chemistry on similar substrates. In addition, a further 10.2% have multiple EC numbers, but all share the same EC subsubclass (i.e., the third level of the EC annotation) and so can be expected to share similar chemistry but on more different substrates. This leaves a further 6.6% that map to multiple EC numbers that differ more fundamentally at the EC class or subclass level. It would make an interesting study to measure and compare the similarity of KEGG reactions mapped to PDB structures via EC numbers or within EC subsubclasses, but this is not considered further here, where the focus remains on the similarity of bound and cognate ligands.

The purpose of this work is not to attempt to assign function to structure based on bound ligands, rather it is intended to prioritize searches from a KEGG or EC perspective to those structures most likely to contain relevant bound ligands. Therefore, this does not eliminate the need for a user to verify that a particular PDB structure can indeed perform the query reaction. Bearing this in mind, it is still informative to compare some of the PDB to KEGG matches above a predefined similarity threshold to manually curated data to provide some insight into the relevance of the best matches.

In order to test the impact of this uncertainty, we have examined a manually curated set of PDBs from the M-CSA (Ribeiro et al., 2017). The M-CSA is a database of enzyme mechanisms, and selects a representative PDB code for each entry using a set of selection criteria, which include best resolution, lack of mutations, and presence of cofactors and/or ligands. We chose to look only at those M-CSA entries that had an associated KEGG reaction with a similarity score of at least 0.7, giving a dataset of 2,629 PDB to KEGG reaction data points. In 84.2% of this dataset, the most similar KEGG reaction from our analysis agreed with the M-CSA manual assignment, meaning that in 15.8% of cases the bound ligands are more similar to a different KEGG reaction, albeit usually a very similar one. This is not necessarily wrong (the enzyme may be promiscuous or the EC nomenclature may be “hierarchical” in some way so that one EC number subsumes another), but this highlights the need to validate the match when the EC number includes more than one KEGG reaction. It should be noted that the number of PDB structures with only one potential KEGG in our M-CSA validation dataset is 45.6%, higher than the overall dataset (38.8%). Looking further down the rankings, the M-CSA manually assigned reaction was in the top two and top five rankings in our analysis for 95.3% and 98.8% of the dataset, respectively. This shows that the manually assigned reaction usually appeared high in the ranking if not always in the top rank, giving confidence that when the similarity score is high, relevant KEGG reactions are being matched.

## Discussion

Our results show that there is high variability in the similarity of bound ligands to cognate ligands across PDB structures, with a high proportion of PDB structures containing no bound ligands or containing ligands with low similarity to the cognate molecule. These results emphasize the importance of a measure of the relevance of the PDB in terms of the similarity of bound and cognate ligands (in addition to other more established metrics such as resolution) to help researchers more easily identify the most biologically relevant PDB structures as starting points for further study.

This variability in similarity of bound and cognate ligands makes some PDB structures much more useful and informative for the diverse virtual screening methods described in the Introduction, which rely on identifying key interactions between ligands and the binding site. Despite the current limitation of low similarity to the cognate ligand in many situations, these virtual screening methods are able to extract relevant information from the bound structure and apply it in a beneficial way in a wide variety of applications. The similarity scores released in the public Mendeley Data Repository and forming the basis of this study will help researchers in the preparation of datasets for these virtual screening methods, circumventing the need to manually review many PDB structures for suitability. We anticipate that this will help in the identification of binding pockets with greater accuracy and specificity and help in the identification of actives when used in virtual screening.

The similarity measure described in this paper finds the proportion of atoms residing in identical topology between molecules, a method we call PARITY. We favor this similarity metric since it avoids the situation where a small change to a crucial connecting atom disrupts the maximum common substructure or molecular paths, having a disproportionately large detrimental effect on the similarity score. It also enables the ligand similarity scores to be conveniently combined into a KEGG reactants/products similarity score favoring more complete matching of reactants of products.

Where there is sufficient similarity between bound and cognate ligands there is potential to use the bound atom coordinates in the matched region from PARITY to constrain the docking of the cognate ligand. The docked cognate-ligand enzyme structures obtained would have the potential to enhance and supplement virtual screening datasets and facilitate the further study of ligand-enzyme interactions. We aim to generate a dataset of docked cognate-ligand enzyme structures to be released alongside the similarity scores in an online database.

## STAR★Methods

### Key Resources Table

**Table:** 

REAGENT or RESOURCE	SOURCE	IDENTIFIER
**Deposited Data**		

ec_pdb_sep17.csv	This paper; Mendeley Data	https://doi.org/10.17632/7c48npgyr8.2#file-d8c87e4a-76b7-4763-bd7d-61a6aa854021
kegg_pdb_sep17.csv	This paper; Mendeley Data	https://doi.org/10.17632/7c48npgyr8.2#file-c3650d9e-36b8-4126-9c57-aa77a5ce92ed
keggCpd_pdb_sep17.csv	This paper; Mendeley Data	https://doi.org/10.17632/7c48npgyr8.2#file-8cd8d174-7b52-4e22-9898-d518c626c798

**Software and Algorithms**		

RDKit	Landrum et al.	http://www.rdkit.org

**Other**		

pdb_chain_enzyme.csv	Protein Data Bank in Europe (PDBe) https://www.ebi.ac.uk/pdbe/	pdb_chain_enzyme.csv.gz https://www.ebi.ac.uk/pdbe/docs/sifts/quick.html
components.cif	Protein Data Bank in Europe (PDBe) https://www.ebi.ac.uk/pdbe/	components.cif ftp://ftp.wwpdb.org/pub/pdb/data/monomers/components.cif
PDB files	Protein Data Bank in Europe (PDBe) https://www.ebi.ac.uk/pdbe/	Not applicable
KEGG compound MOL files KEGG reactions	Kyoto Encyclopedia of Genes and Genomes (KEGG) http://www.genome.jp/kegg/	KEGG API http://www.kegg.jp/kegg/rest/keggapi.html

### Contact for Reagent and Resource Sharing

Further information and requests for resources should be directed to and will be fulfilled by the Lead Contact, Jonathan Tyzack (tyzack@ebi.ac.uk).

### Method Details

The overall goal of this paper is to generate a ranked list of PDB structures according to biological similarity of their ligands. A flow chart describing the different steps in this analysis (explained in more detail below) is presented in Figure 1. At its heart is a method to compare the similarity of bound and cognate ligands, which we call PARITY (Proportion of Atoms Residing in Identical Topology). This measures the similarity of bound ligands in PDB structures (Rose et al., 2015) with cognate ligands from KEGG reactions (Kanehisa et al., 2016), linking via the EC number published in SIFTS (Velankar et al., 2013).

#### Data Set Curation

Firstly, it was necessary to identify enzyme structures within the PDB to form the basis for the similarity study. SIFTS (Structure Integration with Function, Taxonomy and Sequence) (Velankar et al., 2013) is a resource for mapping between PDB and Uniprot, but also consolidates meta data including up-to-date EC references. Therefore, SIFTS was used to obtain the latest mappings of PDB codes to EC references (using the SIFTS file pdb_chain_enzyme.csv dated 2017/09/23). For some structures where only a partial EC reference is listed, the structure was mapped to all downstream leaves in the EC hierarchy.

To be able to carry out the similarity analysis it was necessary to obtain molecular structures for the bound and cognate ligands. Chemical structures for bound PDB ligands were obtained by matching the ligand reference to a database of SMILES strings provided by the PDB (components.cif), but structures for cognate reactants/products are not always uniquely assigned at the EC level. Therefore, the EC references were mapped to KEGG reactions in the KEGG database (Kanehisa, 2000, Kanehisa et al., 2016), enabling PDB structures to be linked to KEGG reaction(s) and chemical structures for cognate ligands to be obtained from MOL file representations within KEGG.

Of the 68,236 PDB structures listed in the SIFTS download, 56,994 could be matched to a KEGG reaction with molecular structures for the reactants/products. Of the remaining 11,242 structures the majority act on polymer substrates: 6,606 belong to EC 3.4 (peptidases); 1,698 belong to EC 3.1 (acting on ester bonds); 1050 belong to EC 3.2 (glycosylases); 838 belong to EC 2.3 acyltransferases; and 1,050 belong to other EC categories. These were excluded from the analysis as similarity calculations are not possible without cognate ligand structures.

KEGG has some generic reactions that contain a Markush structure where the R group represents a position where variation is tolerated and often elucidated in more specific child reactions. The similarity methods described in the next section allow for Markush structures by replacing the R group in the cognate ligand with the matched fragment in the bound ligand, allowing comparable similarity scores to be generated for reactions containing Markush structures.

#### Ligand Similarity Using PARITY

The RDKit cheminformatics toolkit (Landrum, 2016) was used to read molecules in SMILES or MOL file format and perform the similarity calculations. The molecular similarity between bound molecule B and cognate molecule C was calculated using PARITY (Proportion of Atoms Residing in Identical Topology) by identifying the proportion of atoms of the same type residing in identical topological positions in B and C. This is implemented by identifying the maximum common substructure (MCS) in molecules B and C on the most permissive basis, matching any atom and bond type, and then counting the number of atoms of the same type in equivalent positions in each molecule, generating a similarity score using a Tanimoto based formula expressing the intersection over the union:

$$S=\frac{I}{U}=\frac{N_{sim}}{\left(N_{B}+N_{C}-N_{sim}\right)}$$

where *I* represents the intersection of B and C, *U* represents the union of B and C, *N*_*B*_ is the number of atoms in bound ligand B, *N*_*C*_ is the number of atoms in cognate ligand C and *N*_*sim*_ is the number of atoms of the same type in equivalent positons in B and C. The ligand similarity score was binned by rounding to the nearest 0.05 to allow plots of molecule similarity against frequency to be made.

The advantage of this method over simply using the size of the MCS or path based fingerprint methods is the situation where a small change in the center of a molecule disrupts the MCS and causes a disproportionately large negative change in the similarity score. The PARITY method gives a more intuitive and gradual fall in similarity in this situation, as demonstrated by the comparison between C00026 (2-oxoglutarate) and OGA (N-oxalyglycine) in Figure 2 where a difference of just 1 atom retains a relatively high PARITY similarity score of 0.82 but would fall more dramatically to (5/(10 + 10 − 5)) = 0.33 if matching the MCS.

#### Similarity at the KEGG Reactants/Products Level

For each PDB to KEGG comparison, ligand similarity calculations are carried out on an all by all basis comparing all bound ligands to all cognate reactants (i.e. compounds on the left-hand side of the KEGG reaction) and to all cognate products (i.e. compounds on the right-hand side of the KEGG reaction), taking the best match to either reactants or products. Whether the match has been made to either KEGG reactants or products is recorded and explicitly documented in the output data.

To ensure complete matching of the cognate reaction, it is important to note that all cognate reactants/products must be matched to a bound ligand, and any remaining unmatched cognate molecules will appear as unmatched in the final calculation and be fully reflected in the final similarity score. However, due to the presence of water as a solvent in PDB structures and the difficulty of resolving the positions of hydrogen atoms, compounds C00001 (water), C00080 (proton) and C00282 (dihydrogen) were excluded from the cognate ligands. The cognate ligands are matched to their most similar bound ligand using a greedy matching algorithm, i.e. the next most similar pair of unmatched cognate and bound ligands is always matched.

From the resulting list of matched cognate and bound ligands the similarity scores can conveniently be combined to generate a similarity score on a KEGG reactants/products basis. For example, if there are M cognate molecules *C*_*m*=1→*M*_ on one side of a KEGG reaction, and N bound molecules *B*_*n*=1→*N*_ in the PDB, ligand similarity scores are calculated as follows:

$$\begin{array}{c} S_{C_{1}B_{1}}=\frac{I_{C_{1}B_{1}}}{U_{C_{1}B_{1}}}=\frac{N_{sim_{C_{1}B_{1}}}}{\left(N_{C_{1}}+N_{B_{1}}-N_{sim_{C_{1}B_{1}}}\right)}\\ S_{C_{2}B_{2}}=\frac{I_{C_{2}B_{2}}}{U_{C_{2}B_{2}}}=\frac{N_{sim_{C_{2}B_{2}}}}{\left(N_{C_{2}}+N_{B_{2}}-N_{sim_{C_{2}B_{2}}}\right)}\\ \ldots \\ S_{C_{M}B_{M}}=\frac{I_{C_{M}B_{M}}}{U_{C_{M}B_{M}}}=\frac{N_{sim_{C_{M}B_{M}}}}{\left(N_{C_{M}}+N_{B_{M}}-N_{sim_{C_{M}B_{M}}}\right)} \end{array}$$

In the case where M < N any remaining unmatched bound ligands are discarded; better matches to the cognate molecules have been found. In the case where M > N there are not enough bound molecules to match all of the cognate molecules, but the similarity score is still calculated comparing to an empty molecule *B*_0_ as follows:

$$S_{C_{m}B_{0}}=\frac{I_{C_{m}B_{0}}}{U_{C_{m}B_{0}}}=\frac{N_{sim_{C_{m}B_{0}}}}{\left(N_{C_{m}}+N_{B_{0}}-N_{sim_{C_{m}B_{0}}}\right)}=\frac{0}{\left(N_{C_{m}}+0-0\right)}=\frac{0}{N_{C_{m}}}$$

The similarity scores can then be combined to give a reaction similarity score using:

$$S_{reactants/products}=\frac{\sum_{m=1}^{M}I_{C_{m}B_{m}}}{\sum_{m=1}^{M}U_{C_{m}B_{m}}}$$

substituting the expressions from above. In this way, a similarity score is obtained at the KEGG reactants/products level by combining the similarity scores of the best matches at the ligand level.

The methodology to calculate similarity at the KEGG reactants/products level is demonstrated for PDB 3AVR (Catalytic fragment of UTX/KDM6A bound with histone H3K27me3 peptide, N-oxyalylglycine, and Ni(II)) and KEGG R05493 (alpha-ketoglutarate-dependent 2,4-dichlorophenoxyacetate dioxygenase) in Figure 2.

#### Rank Ordered Similarity for Each KEGG and EC

Once the similarity scores have been calculated the PDB-KEGG comparisons were rank ordered to identify the PDB structures containing the most representative ligands for each EC reference and each KEGG reaction. A Mendeley data repository contains 2 files (ec_pdb_sep17.csv and kegg_pdb_sep17.csv) with the top ranked matches for each EC and KEGG respectively, along with any other PDB-KEGG matches with a similarity score greater than or equal to 0.70. A breakdown of each PDB-KEGG match by pairs of matched bound-cognate ligands is given in the final column. However, to facilitate easy searching by KEGG compound we include an additional file (keggCpd_pdb_sep17.csv) showing for each KEGG compound in each KEGG reaction, rank ordered matches to PDB files where similarity to a bound ligand is greater than or equal to 0.7.

The content of the PDB reflects the areas of focus for structural biologists and contains many duplicate structures and homologues, so PDBs were clustered based on identical Fasta sequence to enable the best PDB structure to be identified for each cluster.

### Data and Software Availability

The csv files described in Rank ordered similarity for each KEGG and EC in Method details have been made available in a Mendeley data repository that can be accessed on https://data.mendeley.com/datasets/7c48npgyr8:

ec_pdb_sep17.csv https://doi.org/10.17632/7c48npgyr8.2#file-d8c87e4a-76b7-4763-bd7d-61a6aa854021

kegg_pdb_sep17.csv https://doi.org/10.17632/7c48npgyr8.2#file-c3650d9e-36b8-4126-9c57-aa77a5ce92ed

keggCpd_pdb_sep17.csv https://doi.org/10.17632/7c48npgyr8.2#file-8cd8d174-7b52-4e22-9898-d518c626c798
